# Rasagiline protects against alpha-synuclein induced sensitivity to oxidative stress in dopaminergic cells

**DOI:** 10.1016/j.neuint.2010.06.017

**Published:** 2010-11

**Authors:** K.Y. Chau, J.M. Cooper, A.H.V. Schapira

**Affiliations:** Department of Clinical Neurosciences, UCL Institute of Neurology, Rowland Hill St., London NW3 2PF, United Kingdom

**Keywords:** Parkinson's disease, Alpha-synuclein, Rasagiline, Mitochondria, Free radicals, Neuroprotection

## Abstract

Rasagiline is a propargylamine and irreversible monoamine oxidase (MAO) B inhibitor used for the treatment of Parkinson's disease (PD). It has demonstrated neuroprotective properties in laboratory studies. Current concepts of PD aetiopathogenesis include the role of alpha-synuclein, protein aggregation, free radical metabolism and mitochondrial dysfunction in contributing to cell death. We have used a combination of alpha-synuclein and free radical mediated toxicity in a dopaminergic cell line to provide a model of nigral toxicity in order to investigate the potential molecular mechanisms that mediate rasagiline protection. We demonstrate that rasagiline protects against cell death induced by the combination of free radicals generated by paraquat and either wild-type or A53T mutant alpha-synuclein over-expression. This protection was associated with a reduction in caspase 3 activation, a reduction in superoxide generation and a trend to ameliorate the fall in mitochondrial membrane potential. Rasagiline induced an increase in cellular glutathione levels. The results support a role for rasagiline in protecting dopaminergic cells against free radical mediated damage and apoptosis in the presence of alpha-synuclein over-expression. The data are of relevance to the interpretation of the potential mechanisms of action of rasagiline in explaining the results of disease modification trials in PD.

## 1 Introduction

Parkinson's disease (PD) is a progressive multi-centric neurodegenerative disease whose predominant early diagnostic features are secondary to the loss of nigrostriatal dopaminergic neurons. The current concepts of aetiopathogenesis of PD incorporate genetic and environmental influences (Schapira, 2006, 2008a). Alpha-synuclein appears to be important in both genetic and sporadic forms of PD. Point mutations, e.g. A53T, A30P and multiplications have been identified as causes of familial PD, and in the latter the level of protein expression relates to age of onset: the greater the expression, the younger the onset of PD (Fuchs et al., 2007). Polymorphisms of the alpha-synuclein promoter region that increase protein expression enhance the risk for the PD (Ross et al., 2008). The observation that alpha-synuclein is a major constituent of Lewy bodies, indicates its importance in the pathology of sporadic PD. The mechanisms by which alpha-synuclein toxicity are mediated are not fully understood, but are thought to include free radical mediated damage, mitochondrial dysfunction and the promotion of cell death by apoptosis (Schapira, 2006).

Rasagiline is a propargylamine and an irreversible inhibitor of monoamine oxidase B (MAOB). This compound has demonstrated protective properties in vitro against a number of dopaminergic toxins including the nitric oxide donor 3-morpholinosydnoniminehydrochloride (SIN-1), glutamate, 6-hydroxydopamine (6-OHDA), 1-methyl-4-phenyl pyridinium (MPP^+^), β-amyloid, tetrahydroisoquinoline, and serum and growth factor deprivation (for reviews see Mandel et al., 2005; Schapira, 2008b). This protection is not dependent upon the MAOB inhibitory action of rasagiline as the MAO inactive S-enantiomer of rasagiline is as potent in protection studies as the active form, and protection is seen in cells that do not express MAOB (Maruyama et al., 2003; Youdim et al., 2001a,b; Youdim and Weinstock, 2001). Rasagiline exerts its protection in certain of these models via an anti-apoptotic action that may be mediated by activation of Bcl-2 and Bcl-xL and the protein kinase C/mitogen-activated protein kinase (PKC/MAPK) pathway, and down-regulation of pro-apoptotic molecules such as Bax and Bad (Mandel et al., 2005; Youdim et al., 2005a,b). Aminoindan, the major metabolite of rasagiline has no MAOB inhibitory action but has been reported to exert some protective effects (Bar-Am et al., 2007; Chen et al., 2007).

Drugs currently available for the treatment of PD are predominantly directed to enhancing dopaminergic function and providing relief of motor symptoms. Considerable effort is now focussed on developing compounds that will slow or stop progression of neurodegeneration (Olanow et al., 2008; Schapira, 1999). We hypothesized that given the actions of rasagiline in protecting against dopaminergic toxins, this molecule would be protective against the dopamine cell toxicity associated with alpha-synuclein.

## 2 Materials and methods

### 2.1 Cell culture

SHSY5Y cells were cultured in a 1:1 mixture of Dulbecco's modified Eagle's medium and Ham's F12 medium supplemented with 10% fetal bovine serum (Labtech, East Sussex, UK), 0.01 μM nonessential amino acids, 100 U/ml penicillin and 100 μg/ml streptomycin (all from Invitrogen, San Diego, CA), at 37 °C under humidified 5% CO_2_ atmosphere. Two SHSY5Y cell lines each stably over-expressing similar levels of wild-type (WTSyn) or mutant (A53TSyn) alpha-synuclein, and the controlled cells containing the empty expression plasmid, were maintained in 400 μg/ml G418 (PAA labs, Pasching, Austria), and their characterisation has been previously described (Chau et al., 2009).

### 2.2 Chemicals

Paraquat, rotenone, Triton X-100, and H_2_ were purchased from Sigma–Aldrich (St. Louis, MO). Rasagiline was provided by Teva.

### 2.3 Treatments

**Fig. 1:**
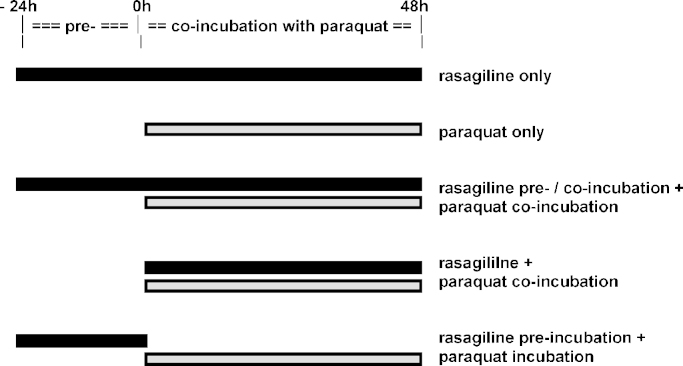
Schematic illustration on the various combinations of incubation between rasagiline and paraquat.

The incubation schedules of rasagiline and paraquat are illustrated in Fig. 1.

### 2.4 Measurement of cell death (LDH assays)

Percentage release of lactate dehydrogenase (LDH) was measured by the Promega 96 CytoTox assay kit (Madison, WI). In brief, SHSY5Y cells were seeded into 24-well plates and left to grow to 60% confluence, achieved typically within 48 h from seeding at 40%. The medium was replaced by culture medium lacking -red. After incubations the medium was measured for LDH release, whereas in a duplicate well cells were lysed by the addition of Triton X-100 (to a final of 1%) to measure total LDH. After mixing the samples with the substrate and incubated at room temperature for 30 min, OD was measured using a plate reader (Biorad, Hercules, CA) with filter of 492 nm. The percentage of LDH release was calculated from the medium and total values corrected for background.

### 2.5 Measurement of capase-3 activity

SHSY5Y cells grown on 12-well plates were incubated in phenol-red-free medium. Cells collected were lysed according to the manufacturer's instruction and protein level of lysates determined by BCA protein assays kit (Pierce, Rockford, IL). Lysates of equal amount of protein were mixed with reaction buffer and caspase-3 substrate Z-DEVD-R110 (Molecular Probes, Eugene, OR) into a 96-well plate incubated at room temperature for 1 h and read using a fluorescence plate reader (Bio-Tek's Synergy HT model, Winooski, VT), with excitation (Ex) and emission (Em) wavelengths of 485 ± 20 nm and 528 ± 20 nm.

### 2.6 Measurement of mitochondrial membrane potential (TMRM staining)

To SHSY5Y cells grown on 24-well plates was added tetramethyl rhodamine methyl ester (25 μM Molecular Probes), and incubated at 37 °C for 30 min. Attached cells were washed and the TMRM mitochondrial staining was quantified by a scanning function of 3 × 3 matrix to the bottom of the well, using the Synergy HT microplate reader set as 530 ± 25 nm Ex/590 ± 35 nm Em. To a duplicate well treated in the same manner, total LDH was determined as above and this reading was used to correct for cell number.

### 2.7 Measurement of reactive oxygen species (DHE staining)

SHSY5Y cells seeded onto 24-well plates were stained with growth medium without FBS containing 20 μM dihydroethidium (DHE, Molecular Probes) for 30 min at 37 °C. Adherent cells were washed and intracellular ethidium (oxidised product of DHE), was measured by scanning the bottom of the well by a 5 × 5 matrix, using the Synergy HT microplate reader with an excitation and emission wavelength of 530 ± 25 nm and 590 ± 35 nm. The DHE taken up by the cells was fully oxidised by addition of 88 mM H_2_O_2_ and 50 μg/ml peroxidase in the presence of 1% Triton X-100 and further incubated at 37°C for 1 h, before measuring fluorescence again. Percentage oxidised DHE represents the percentage of oxidised DHE over total DHE taken up.

### 2.8 Measurement of glutathione content

Intracellular glutathione concentration was measured by a kit provided by Calbiochem (San Diego, CA), using a metaphosphoric acid lysate of SHSY5Y cells collected from a 10 cm plate. BCA protein assay was performed on the lysate to correct for protein content.

### 2.9 Statistical analysis

Independent experimental repeats were carried out as indicated, and the results are presented as mean values ± standard error of mean (error bars). Unpaired t test, one-way ANOVA followed by Dunnett or Tukey–Kramer's post-test analysis was performed. A *p* value of ≤0.05 was considered statistically significant.

## 3 Results

### 3.1 Rasagiline reduced cell death induced by paraquat and alpha-synuclein over-expression

**Fig. 2:**
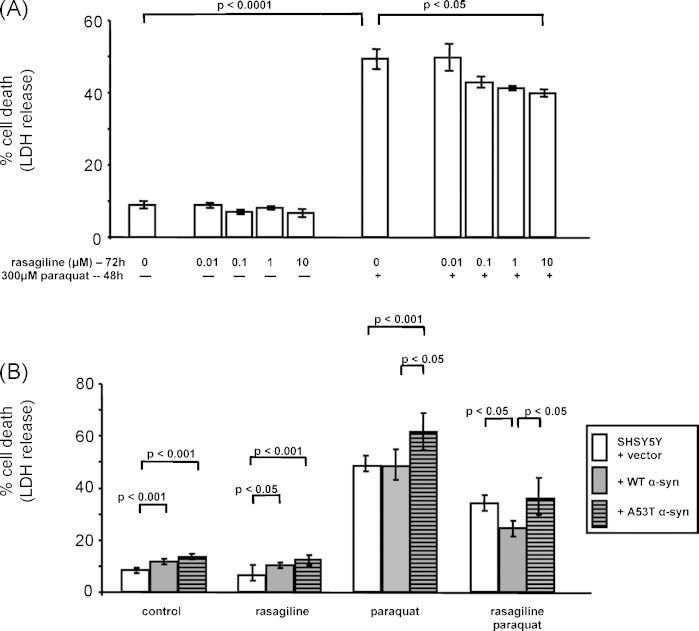
Rasagiline reduced death induced by paraquat in SHSY5Y cells, and cells over-expressing wild-type and A53T mutation of alpha-synuclein. Paraquat exposure for 48 h in (A) normal SHSY5Y cells, and (B) 2 independent cell lines each expressing similar levels of wild-type (WT) and mutant alpha-synuclein (A53T), and the control SHSY5Y cells harbouring the empty expression plasmid. Cell death measured by LDH release and expressed as mean ± standard error of mean, of at least 6 experimental repeats. Data of both cell lines from each model are combined. Significance determined by unpaired t test, one-way ANOVA followed by Dunnett and Tukey–Kramer's post-test accordingly.

Incubation of the free radical inducing agent paraquat (300 μM) for 48 h induced a significant increase in cell death in SHSY5Y cells (from 9% to 49%, *p* < 0.0001, 22 degrees of freedom, *F* = 13.033 by unpaired t test, Fig. 2A). When rasagiline (0–10 μM) was added 24 h before (pre-incubation) and for 48 h with paraquat (co-incubation) cell death was reduced in a dose dependent manner, reaching significance at 10 μM with a reduction of 19% (*p* < 0.05, 4 degrees of freedom between treatments and 45 degree of freedom between residuals, *F* = 3.574, by one-way ANOVA followed by Dunnett's post-test, Fig. 2A). Pre-incubation or co-incubation of rasagiline alone did not decrease cell death (data not shown). Based upon these preliminary studies, 10 μM rasagiline and 300 μM paraquat were used in all further studies. The dose of paraquat selected was based upon its ability to induce moderate cell death at a level that could potentially be reversed by a protective agent.

Stable over-expression of either wild-type (WTSyn) or mutant (A53TSyn) alpha-synuclein in SHSY5Y cells resulted over a short time scale (48–72 h) in small but significant increases in cell death under basal conditions (both *p* < 0.001, 2 degrees of freedom between treatments and 65 degree of freedom between residuals, *F* = 18.934, by one-way ANOVA followed by Tukey–Kramer's post-test, Fig. 2B). The addition of 10 μM rasagiline for 72 h had no effect on baseline cell death in these cells (Fig. 2B). Incubation with 300 μM paraquat over 48 h caused a significant increase in death in all cell lines tested (*p* < 0.001 for all lines relative to untreated cells; 3 degrees of freedom between treatments and 101 degree of freedom between residuals, *F* = 78.392 for control cells; 3 degrees of freedom between treatments and 77 degree of freedom between residuals, *F* = 77.313 for WTSyn cells; 3 degrees of freedom between treatments and 100 degree of freedom between residuals, *F* = 67.985 for A53TSyn cells; all by one-way ANOVA followed by Tukey–Kramer's post-test). The A53TSyn cells were significantly more sensitive to the paraquat treatment compared to both control SHSY5Y and WTSyn cells (*p* < 0.001 and 0.05 respectively, 2 degrees of freedom between treatments and 99 degree of freedom between residuals, *F* = 8.429, by one-way ANOVA followed by Tukey–Kramer's post-test, Fig. 2B). Rasagiline (10 μM pre- and co-incubation) significantly decreased the mean cell death induced by paraquat treatment in all cell lines (by 19%, 49% and 42% in control, WTSyn and A53TSyn cells respectively, all *p* < 0.001; degrees of freedom and *F* values as above after one-way ANOVA followed by Tukey–Kramer's post-test), such that death in the A53TSyn cells was now comparable to the other lines (Fig. 2B).

### 3.2 Rasagiline decreased paraquat-induced caspase-3 activation

**Fig. 3:**
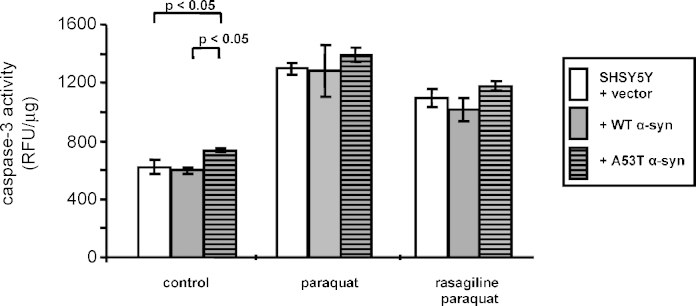
Rasagiline decreased caspase-3 activated by paraquat exposure in empty vector SHSY5Y cells and cells expressing wild-type or A53T alpha-synuclein. Caspase-3 activity was measured by cleavage of Z-DEVD-R110 to release fluorescence that was corrected for protein level. Shown is the mean fluorescence ± standard error of mean, of 9 experiments. One-way ANOVA followed by Tukey–Kramer's post-test was carried out to compare between treatments.

Caspase-3 activity under basal conditions was significantly greater in the mutant A53TSyn cells compared to both control and WT SHSY5Y lines (both *p* < 0.05, 2 degrees of freedom between treatments and 61 degree of freedom between residuals, *F* = 4.812, by one-way ANOVA followed by Tukey–Kramer's post-test, Fig. 3). Caspase-3 activity was increased by exposure to paraquat in control, WTSyn and A53TSyn expressing SHSY5Y cells (*p* > 0.001 for all 3 groups; 3 degrees of freedom between treatments and 100 degrees of freedom between residuals, *F* = 119.14 for control cells; 3 degrees of freedom between treatments and 56 degrees of freedom between residuals, *F* = 33.679 for WTSyn cells; 3 degrees of freedom between treatments and 73 degree of freedom between residuals, *F* = 22.205 for A53TSyn cells; all by one-way ANOVA followed by Tukey–Kramer's post-test) with no significant difference between the lines. Rasagiline treatment (10 μM pre- and co-incubation) significantly reduced the paraquat-induced caspase activity in the control and WTSyn expressing SHSY5Y cells (by 15%, *p* < 0.001 and 21%, *p* < 0.05 respectively; degrees of freedom and *F* values as above after one-way ANOVA followed by Tukey–Kramer's post-test). A 15% reduction was observed in A53TSyn lines although this did not reach statistical significance.

**Fig. 4:**
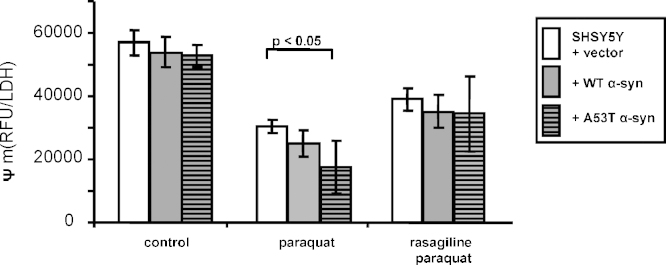
Rasagiline effect on mitochondrial membrane potential (*Ψ*_m_) reduction caused by paraquat in empty vector SHSY5Y cells and cells expressing wild-type or A53T alpha-synuclein. Mitochondrial membrane potential was measured by TMRM staining and corrected for cell number by total LDH level. Mean TMRM signal ± standard error of mean of 5 experiments, is illustrated. One-way ANOVA followed by Tukey–Kramer's post-test was carried out by comparing between treatments.

Mitochondrial membrane potential was similar in all the untreated cell lines (Fig. 4). Following paraquat treatment the membrane potential decreased in all lines, but with most reduction seen in the mutant A53TSyn cells (to 67% in A53TSyn; 47% in control and 54% in WTSyn, all *p* > 0.001; 3 degrees of freedom between treatments and 81 degree of freedom between residuals, *F* = 10.339 for control cells; 3 degrees of freedom between treatments and 81 degrees of freedom between residuals, *F* = 14.08 for WTSyn cells; 3 degrees of freedom between treatments and 45 degree of freedom between residuals, *F* = 7.323 for A53TSyn cells; all by one-way ANOVA followed by Tukey–Kramer's post-test). The reduced membrane potential seen in the A53TSyn cells was significantly greater than in control cells (*p* < 0.05, 2 degrees of freedom between treatments and 50 degree of freedom between residuals, *F* = 3.283, by one-way ANOVA followed by Tukey–Kramer's post-test, Fig. 4). There was a trend for the addition of 10 μM rasagiline to restore the membrane potential in all cells, but this did not reach statistical significance. However, there was also no longer any significant difference in the membrane potential between control and A53TSyn cells (Fig. 4).

### 3.3 Rasagiline reduced free radical generation and increased glutathione levels

**Fig. 5:**
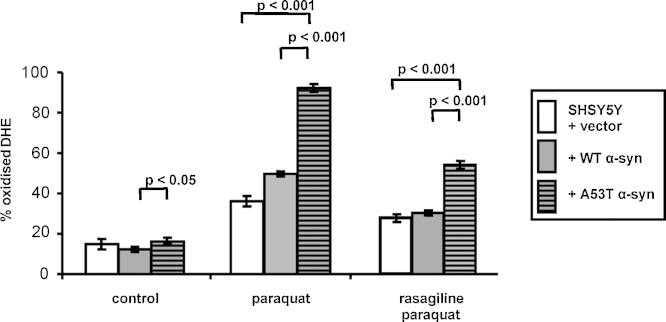
Rasagiline effect on the oxidation of dihydroethidium (DHE) as a measurement of reactive oxygen species induced by paraquat in empty vector SHSY5Y cells and cells expressing wild-type or A53T alpha-synuclein. Oxidised DHE, followed by the total DHE (after addition of peroxidase, H_2_O_2_ and Triton X-100), was measured and expressed as % oxidised DHE, of the mean ± standard error of mean of 8 experiments. One-way ANOVA followed by Tukey–Kramer's post-test was used to compare treatments.

In untreated cells, there was a small but significant increase in free radical generation as measured by DHE in A53TSyn cells compared to WTSyn cells, but not compared to controls (*p* < 0.05, 2 degrees of freedom between treatments and 100 degree of freedom between residuals, *F* = 4.312, by one-way ANOVA followed by Tukey–Kramer's post-test, Fig. 5). Paraquat treatment induced a significant increase in superoxide production (increased by 142%, 306% and 462% in control, WTSyn and A53TSyn cells respectively, all *p* < 0.001; 3 degrees of freedom between treatments and 122 degree of freedom between residuals, *F* = 88.774 for control cells; 3 degrees of freedom between treatments and 140 degree of freedom between residuals, *F* = 122.87 for WTSyn cells; 3 degrees of freedom between treatments and 140 degree of freedom between residuals, *F* = 406.55 for A53TSyn cells; all by one-way ANOVA followed by Tukey–Kramer's post-test) compared to the respective untreated cell lines. Furthermore, the increase in DHE oxidation in A53TSyn cells was significantly greater than that seen in either control or WTSyn cells (both *p* < 0.001, 2 degrees of freedom between treatments and 101 degree of freedom between residuals, *F* = 177.43, by one-way ANOVA followed by Tukey–Kramer's post-test, Fig. 5). Rasagiline (10 μM pre- and co-incubation) reduced DHE oxidation in all 3 lines (by 22%, 40% and 41% in control, WTSyn and A53TSyn cells respectively, all *p* < 0.001 compared to paraquat alone; degrees of freedom and *F* values as above after one-way ANOVA followed by Tukey–Kramer's post-test). However, although significantly reduced in the presence of rasagiline, DHE oxidation was still significantly higher in the A53TSyn cells compared to the control and WT cells (both *p* < 0.001, 2 degrees of freedom between treatments and 101 degree of freedom between residuals, *F* = 54.5, by one-way ANOVA followed by Tukey–Kramer's post-test, Fig. 5).

**Fig. 6:**
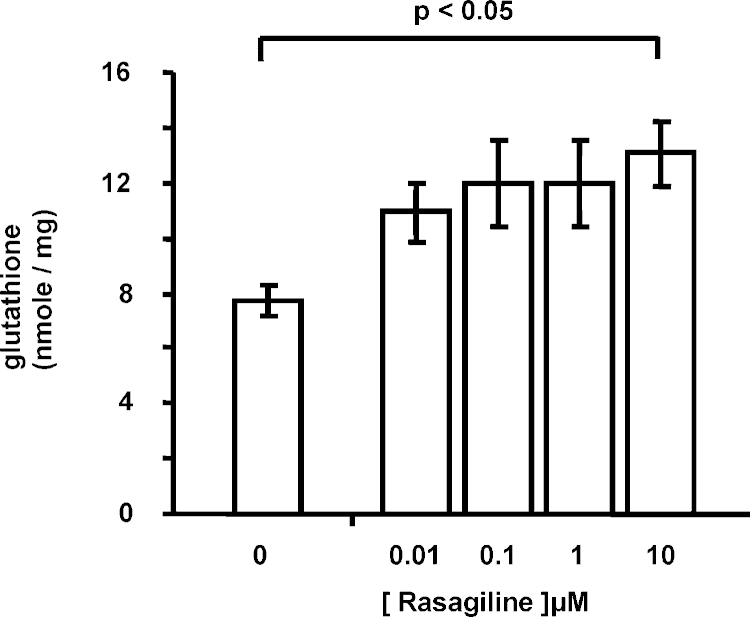
Influence of rasagiline on glutathione levels in SHSY5Y cells. SHSY5Y cells were incubated with increasing level of rasagiline for 24 h and glutathione content was measured that was corrected for protein level. Shown is a summary of 7 experiments its mean ± standard error of mean (significance determined by ANOVA followed by Dunnett post-test).

Incubation of rasagiline for 24 h was found to increase glutathione levels significantly in SHSY5Y cells (*p* < 0.05, 4 degrees of freedom between treatments and 38 degree of freedom between residuals, *F* = 2.684, by one-way ANOVA followed by Dunnett's post-test; Fig. 6).

## 4 Discussion

Alpha-synuclein is the major protein component of Lewy bodies and Lewy neurites in PD brain (Spillantini et al., 1997) and both point mutations and multiplications of the alpha-synuclein gene, as well as polymorphisms that increase expression, have been associated with familial PD (Fuchs et al., 2007; Ross et al., 2008). Conversely, deletion of alpha-synuclein in mice protects dopaminergic neurons against both 6-hydroxydopamine and MPTP (1-methyl-4-phenyl-1,2,3,6-tetrahydropyridine) toxicity (Dauer et al., 2002; Varez-Fischer et al., 2008). Several studies support a role for oxidative and/or nitrative stress in alpha-synuclein modification and/or aggregation (Giasson et al., 2000). We have previously shown that increased expression of A53T mutant alpha-synuclein increases susceptibility to dopamine toxicity, potentially by increasing cytosolic dopamine (Tabrizi et al., 2000). A recent report has demonstrated that a combination of increased WT alpha-synuclein expression, elevated cytoplasmic calcium and dopamine levels enhances selective dopaminergic cell death (Mosharov et al., 2009). Thus, our model incorporating both free radical generation and alpha-synuclein expression provides a useful tool to investigate the interaction of pathogenetic pathways in PD and potential targets for neuroprotection. We have recently used this model to show that the dopamine receptor agonist cabergoline is able to protect against alpha-synuclein toxicity, an effect partially mediated via dopamine receptors (Chau et al., 2009).

Rasagiline is a propargylamine, one of a group of molecules considered to have neuroprotective potential in PD (Schapira, 2008a). The propargylamine selegiline has demonstrated neuroprotective properties in vitro, in vivo and possibly in PD patients although there remains debate regarding its efficacy in the latter (The Parkinson Study Group, 1993). It is notable that selegiline is metabolised to amphetamines which can themselves inhibit the protective action of the parent molecule (Bar et al., 2004; Bar-Am et al., 2004). TCH346, another propargylamine was protective in numerous pre-clinical models of PD, but failed to demonstrate any protective effect in PD patients, although this may reflect errors in clinical trial design (Olanow et al., 2006). Rasagiline is metabolised to aminoindan, which may itself have protective properties through anti-apoptotic actions (Bar-Am et al., 2007). Rasagiline has demonstrated the capacity to protect a variety of neuronal types against a range of toxic processes in vitro and in animal models (Mandel et al., 2005). We have explored the possible mechanisms of action of rasagiline that might mediate a protective action against cell death in a cell model that combines important features of PD pathogenesis.

In our SHSY5Y WTSyn and A53TSyn stable over-expression models, there was an increase in cell death compared to control even over the relatively short time course of 48 h. This innate toxicity of alpha-synuclein expression has been seen in a variety of cell lines and animal models (reviewed in Olanow and Kordower, 2009). Paraquat caused a significant increase in death in all our cell lines accompanied by increased free radical generation, reduced mitochondrial membrane potential and increased caspase activation. The A53T mutant cells were significantly more sensitive to paraquat, indicating a synergistic and detrimental action of free radicals in these cells. The explanation for this synergistic effect on toxicity is not certain, but several studies have suggested that oxidative stress causes accumulation alpha-synuclein protofibrils (Conway et al., 2001) and promotes oligomerisation (Norris et al., 2003).

Rasagiline was able to reduce cell death in all lines in this combined toxicity model. The reduction in caspase-3 activation and the trend to protect against a fall in mitochondrial membrane potential are both in line with an action of rasagiline via an inhibition of mitochondria-mediated apoptosis. This is in agreement with previous studies on rasagiline (Mandel et al., 2005). Furthermore, we demonstrated that rasagiline can increase cellular GSH levels and reduce superoxide generation in the paraquat–alpha-synuclein model, suggesting that its protective actions incorporate an anti-oxidant effect. The protective actions of rasagiline were such that it was able to counter the additional sensitivity to paraquat conferred by the expression of A53T alpha-synuclein.

These in vitro studies confirm a protective action of rasagiline against a free radical generator and extend its potential efficacy to a combined genetic-toxin model: WT or mutant alpha-synuclein over-expression. Our results support the concept that free radical generation can enhance alpha-synuclein toxicity and show for the first time that rasagiline can ameliorate this effect. It is of interest that alpha-synuclein toxicity may be propagated beyond the host cell (Desplats et al., 2009; Kordower et al., 2008; Li et al., 2008) and it is possible that this may be enhanced in an oxidative environment. The relevance of these observations to modification of the course of PD by rasagiline remains uncertain.

The in vitro and in vivo protective actions of rasagiline have led to it being tested as a drug that might have potential to modify the clinical course of PD. Rasagiline confers symptomatic benefit in PD patients by virtue of its monoamine oxidase B inhibition (Parkinson Study Group, 2004). The results of the ADAGIO^3^ (attenuation of disease progression with rasagiline once-daily) have recently been reported (Olanow et al., 2009). The 1 mg dose met a series of three hierarchical end points including slowing the rate of deterioration in motor function in those randomised to drug versus placebo, a better motor outcome at 18 months and no convergence of the rates of progression in the second 9 months of the study.

The interpretation of the ADAGIO results is complex (Olanow et al., 2009; Schapira, 2009) but include *inter alia* the possibility of a true neuroprotective effect. The ability of rasagiline to protect against paraquat–alpha-synuclein toxicity in our model, at least in part through anti-oxidant and anti-apoptotic roles, is of interest in this context. Our results cannot confirm or support a neuroprotective role for rasagiline in PD patients, but do nevertheless provide a potential mechanism of action for a putative protective effect.

## Conflicts of interest

A.H.V. Schapira has received honoraria for contributions to educational symposia and for the provision of advice in the development of rasagiline.

## References

[bib1] Bar A.O., Amit T., Youdim M.B. (2004). Contrasting neuroprotective and neurotoxic actions of respective metabolites of anti-Parkinson drugs rasagiline and selegiline. Neurosci. Lett..

[bib2] Bar-Am O., Amit T., Youdim M.B. (2007). Aminoindan and hydroxyaminoindan, metabolites of rasagiline and ladostigil, respectively, exert neuroprotective properties in vitro. J. Neurochem..

[bib3] Bar-Am O. (2004). Regulation of protein kinase C by the anti-Parkinson drug, MAO-B inhibitor, rasagiline and its derivatives, in vivo. J. Neurochem..

[bib4] Chau K.Y. (2009). Protection against paraquat and A53T alpha-synuclein toxicity by cabergoline is partially mediated by dopamine receptors. J. Neurol. Sci..

[bib5] Chen J.J., Swope D.M., Dashtipour K. (2007). Comprehensive review of rasagiline, a second-generation monoamine oxidase inhibitor, for the treatment of Parkinson's disease. Clin. Ther..

[bib6] Conway K.A. (2001). Kinetic stabilization of the alpha-synuclein protofibril by a dopamine–alpha-synuclein adduct. Science.

[bib7] Dauer W. (2002). Resistance of alpha-synuclein null mice to the Parkinsonian neurotoxin MPTP. Proc. Natl. Acad. Sci. U.S.A..

[bib8] Desplats P. (2009). Inclusion formation and neuronal cell death through neuron-to-neuron transmission of alpha-synuclein. Proc. Natl. Acad. Sci. U.S.A..

[bib9] Fuchs J. (2007). Phenotypic variation in a large Swedish pedigree due to SNCA duplication and triplication. Neurology.

[bib10] Giasson B.I. (2000). Oxidative damage linked to neurodegeneration by selective alpha-synuclein nitration in synucleinopathy lesions. Science.

[bib11] Kordower J.H. (2008). Lewy body-like pathology in long-term embryonic nigral transplants in Parkinson's disease. Nat. Med..

[bib12] Li J.Y. (2008). Lewy bodies in grafted neurons in subjects with Parkinson's disease suggest host-to-graft disease propagation. Nat. Med..

[bib13] Mandel S. (2005). Mechanism of neuroprotective action of the anti-Parkinson drug rasagiline and its derivatives. Brain Res. Brain Res. Rev..

[bib14] Maruyama W. (2003). Anti-apoptotic action of anti-Alzheimer drug, TV3326 [(N-propargyl)-(3R)-aminoindan-5-yl]-ethyl methyl carbamate, a novel cholinesterase-monoamine oxidase inhibitor. Neurosci. Lett..

[bib15] Mosharov E.V. (2009). Interplay between cytosolic dopamine, calcium, and alpha-synuclein causes selective death of substantia nigra neurons. Neuron.

[bib16] Norris E.H. (2003). Effects of oxidative and nitrative challenges on alpha-synuclein fibrillogenesis involve distinct mechanisms of protein modifications. J. Biol. Chem..

[bib17] Olanow C.W. (2006). TCH346 as a neuroprotective drug in Parkinson's disease: a double-blind, randomised, controlled trial. Lancet Neurol..

[bib18] Olanow C.W., Kieburtz K., Schapira A.H. (2008). Why have we failed to achieve neuroprotection in Parkinson's disease?. Ann. Neurol..

[bib19] Olanow C.W., Kordower J.H. (2009). Modeling Parkinson's disease. Ann. Neurol..

[bib20] Olanow C.W. (2009). A double-blind, delayed-start trial of rasagiline in Parkinson's disease. N. Engl. J. Med..

[bib21] Parkinson Study Group (1993). Effects of tocopherol and deprenyl on the progression of disability in early Parkinson's disease. N. Engl. J. Med..

[bib22] Parkinson Study Group (2004). A controlled, randomized, delayed-start study of rasagiline in early Parkinson disease. Arch. Neurol..

[bib23] Ross O.A. (2008). Genomic investigation of alpha-synuclein multiplication and Parkinsonism. Ann. Neurol..

[bib24] Schapira A.H. (1999). Science, medicine, and the future: Parkinson's disease. BMJ.

[bib25] Schapira A.H. (2006). Etiology of Parkinson's disease. Neurology.

[bib26] Schapira A.H. (2008). Mitochondria in the aetiology and pathogenesis of Parkinson's disease. Lancet Neurol..

[bib27] Schapira A.H. (2008). Rasagiline in neurodegeneration. Exp. Neurol..

[bib28] Schapira A.H. (2009). Molecular and clinical pathways to neuroprotection of dopaminergic drugs in Parkinson disease. Neurology.

[bib29] Spillantini M.G., Schmidt M.L., Lee V.M., Trojanowski J.Q., Jakes R., Goedert M. (1997). Alpha-synuclein in Lewy bodies. Nature.

[bib30] Tabrizi S.J. (2000). Expression of mutant alpha-synuclein causes increased susceptibility to dopamine toxicity. Hum. Mol. Genet..

[bib31] Varez-Fischer D. (2008). Modelling Parkinson-like neurodegeneration via osmotic minipump delivery of MPTP and probenecid. J. Neurochem..

[bib32] Youdim M.B. (2001). Rasagiline [N-propargyl-1R(+)-aminoindan], a selective and potent inhibitor of mitochondrial monoamine oxidase B. Br. J. Pharmacol..

[bib33] Youdim M.B. (2001). The anti-Parkinson drug rasagiline and its cholinesterase inhibitor derivatives exert neuroprotection unrelated to MAO inhibition in cell culture and in vivo. Ann. N. Y. Acad. Sci..

[bib34] Youdim M.B., Weinstock M. (2001). Molecular basis of neuroprotective activities of rasagiline and the anti-Alzheimer drug TV3326 [(N-propargyl-(3R)aminoindan-5-YL)-ethyl methyl carbamate]. Cell Mol. Neurobiol..

[bib35] Youdim M.B., Maruyama W., Naoi M. (2005). Neuropharmacological, neuroprotective and amyloid precursor processing properties of selective MAO-B inhibitor antiParkinsonian drug, rasagiline. Drugs Today (Barc.).

[bib36] Youdim M.B. (2005). Rasagiline: neurodegeneration, neuroprotection, and mitochondrial permeability transition. J. Neurosci. Res..

